# Machine Learning for Wireless Sensor Networks Security: An Overview of Challenges and Issues

**DOI:** 10.3390/s22134730

**Published:** 2022-06-23

**Authors:** Rami Ahmad, Raniyah Wazirali, Tarik Abu-Ain

**Affiliations:** 1 Institute of Networked and Embedded Systems, University of Klagenfurt, 9020 Klagenfurt, Austria; 2 Ubiquitous Sensing Systems Lab, University of Klagenfurt-Silicon Austria Labs, 9020 Klagenfurt, Austria; 3 College of Computing and Informatics, Saudi Electronic University, Riyadh 11673, Saudi Arabia; t.aboain@seu.edu.sa

**Keywords:** wireless sensor networks, machine learning, WSNs security, 6LoWPAN, ZigBee

## Abstract

Energy and security are major challenges in a wireless sensor network, and they work oppositely. As security complexity increases, battery drain will increase. Due to the limited power in wireless sensor networks, options to rely on the security of ordinary protocols embodied in encryption and key management are futile due to the nature of communication between sensors and the ever-changing network topology. Therefore, machine learning algorithms are one of the proposed solutions for providing security services in this type of network by including monitoring and decision intelligence. Machine learning algorithms present additional hurdles in terms of training and the amount of data required for training. This paper provides a convenient reference for wireless sensor network infrastructure and the security challenges it faces. It also discusses the possibility of benefiting from machine learning algorithms by reducing the security costs of wireless sensor networks in several domains; in addition to the challenges and proposed solutions to improving the ability of sensors to identify threats, attacks, risks, and malicious nodes through their ability to learn and self-development using machine learning algorithms. Furthermore, this paper discusses open issues related to adapting machine learning algorithms to the capabilities of sensors in this type of network.

## 1. Introduction

Wireless Sensor Network (WSN) is one of the most effective methods for many real-time applications, due to its compactness, cost-effectiveness, and ease of deployment [1]. The function of the WSN is to monitor the field of interest, collect the data, and transmit it to the base station (Access point) for post-processing analysis [2]. A large number of sensor nodes are used in some WSN implementations. In addition, these wireless nodes have a limited battery life and memory capacity [3]. Therefore, to obtain the most out of these WSNs, there must be a management system for these WSN nodes capable of regulating the relationship among themselves and with the access point as well.

For example, the ZigBee [4] and 6LoWPAN [5] are two protocols that support management in WSNs developed by the Internet Engineering Team (IETF) for standard transmission over IEEE 802.15.4. These protocols support modern management systems to use IEEE 802.15.4 in the 2.4 GHz band and support short transmission [6]. For example, 6LoWPAN IPv6 provides a connection between WSNs based on IP addresses on different layers. It also uses the 6LoWPAN Low Power and Loss Network (RPL) standard to map the network topology and uses the AES encryption algorithm to secure the WSN connection [7]. However, as the topology of these types of networks is constantly changing, it will have an impact on network routing strategies, delay, multi-layer design, coverage, Quality of Services (QoS), and fault detection [8]. Therefore, it is necessary to reconsider the management of WSNs by designing or incorporating new protocols to deal with the nature of the environments for which these embedded devices are designed.

Security and energy consumption are among the most important challenges in WSNs, as each one negatively affects the other. The increased security complexity of a WSN increases the power consumption of a node and vice versa. Given the challenging environments in which these sensors can operate, the need for both (reducing security and energy consumption) is one of the challenges that recent studies in this field are addressing [9,10]. Furthermore, the use of the traditional methods for providing security, which is known by the Triangle and defined by Confidentiality, Integration, and Authentication (CIA) [11], needs to be reconsidered. The process of encryption of data between two communication devices (two nodes) and associated operations, such as key exchange and encryption, are also considered traditional [12]. Moreover, these technologies are energy-intensive methods, especially as we mentioned in the previous paragraph, in the constant change in network topologies due to the constant movement of WSN nodes. Therefore, finding alternative methods that are simpler and faster is what is being sought. Thus, for example, artificial intelligence algorithms are one of the methods that can be used for this purpose. A node can develop skills to interact with nearby WSN nodes, detect viruses, analyze incoming and outgoing packets, authenticate between nodes, and maintain availability [13].

Machine learning (ML) is one of the most famous branches of artificial intelligence that has been developed, where its algorithms build a mathematical model based on a sample of data [14], known as “training data” to make predictions or decisions without being explicitly programmed to do so [15]. For the reasons listed, the ML nature of WSNs is appropriate: WSN ecosystems are complicated and mathematical frameworks cannot be constructed. Furthermore, some programs use data sets that must be combined to function properly. In addition, WSNs have unexpected dynamics and behaviors, and finally, in line with the nature of WSNs, ML algorithms do not require human intervention [16]. However, there are two main challenges to ML in WSNs: the resources and computational limitations of nodes, and the need for large data sets for learning. As for the security of the WSN networks, one of the most important challenges faced by ML algorithms is the difficulty in applying them to the integrity and confidentiality of security requirements. Therefore, machine learning algorithms can help increase security in wireless networks, reduce all forms of congestion problems [17,18,19], and help authentication processes through the physical layer [20,21,22], and error detection [23,24,25]. Furthermore, ML algorithms have a great advantage in analyzing packets as they travel between WSN nodes and detecting suspicious nodes [26].

Many surveys discussed the role of machine learning algorithms in various fields of wireless sensor networks and the Internet of Things (IoT). For example, authors in [13,16,27] discussed ML algorithms in different WSN applications. Moreover, other authors in [15,28,29,30,31] discussed ML algorithms in sub-domains security, such as congestion traffic and intrusion detection in IoT and WSN. Others discussed security requirements in WSNs, such as [32]. However, none of these reviewed studies discussed the use of ML algorithms to provide security requirements for WSNs in all layers. Therefore, this study provides a detailed description of the security requirements of the WSN and the role of ML algorithms in providing these requirements in all WSN layers. ML algorithms can provide a better method for the security of wireless sensor networks than the traditional methods represented by encryption algorithms. In addition, this paper will discuss the challenges that WSNs will face using ML algorithms and ways to solve them. It also proposes solutions that contribute to the possibility of using ML algorithms in different layers at the same time to ensure the security of wireless sensor networks. Furthermore, it provides a statistical overview of each security framework in WSNs commonly implemented by ML algorithms. The main contributions of this paper can be summarized as follows:

- We explain in detail the security requirements covered by ML algorithms in WSN security in current applications
- We present a systematic and comprehensive survey of current technologies in the literature related to improving the security of WSNs using machine learning techniques. The pros and cons of each technique are also highlighted.
- We describe the limitations of using ML in current security solutions for WSNs, the challenges open to ML algorithms in providing them with security, and future re-search solutions.

**Figure 1:**
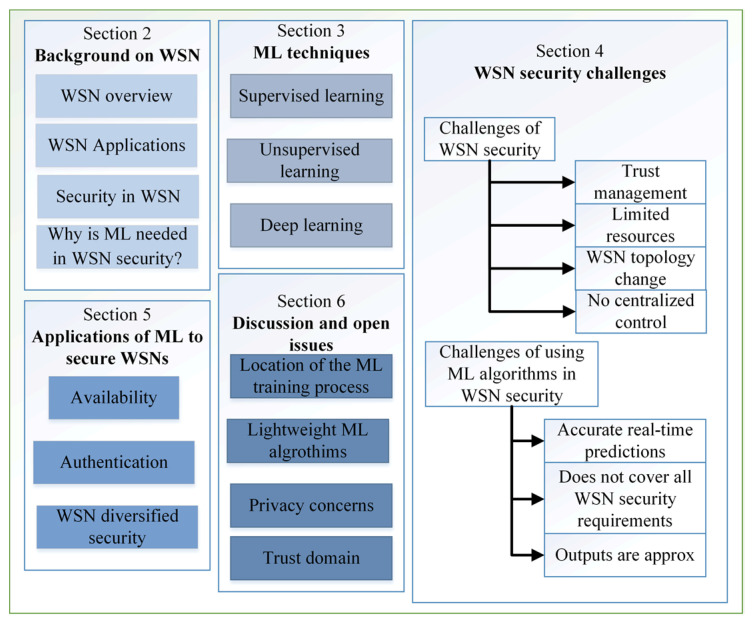
The taxonomy of this survey.

The illustration in Figure 1 shows the scope of this survey and its classification. In Section 2, we first provide a brief background on WSNs infrastructure, then discuss the threats and security requirements. Section 3 discusses the ML algorithms used in WSNs security. Section 4 discusses the security challenges in a wireless sensor network, and Section 5 discusses recent studies that have been developed to mitigate these challenges. After that, open issues related to the security of the WSNs and the role of ML in its future development are discussed in Section 6. Finally, Section 7 concludes the paper.

## 2. Background on WSN

In this part, we discuss the background of the wireless sensor network, as this device is the basic core of the IoT technology concept. This device performs all types of fieldwork, from data collection in all its forms to monitoring, imaging, and other operations. Thus, in this section, we discuss their types, currency method, limitations, and data security through them.

### 2.1. WSN Overview

**Figure 2:**
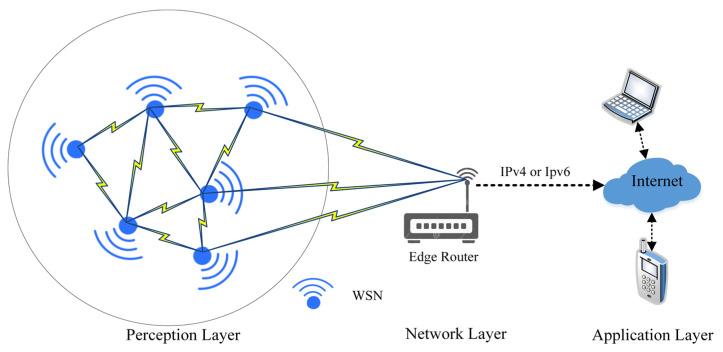
The communication among the WSN layers.

The primary advantage of the IoT is global awareness, intelligent processing, and the reliable transfer of information. The key is the realization of the information’s interactions between a human and a device or device-to-device. These devices consist of embedded systems, control and automation systems, WSNs, and others that share information in different environments for enabling the IoT [33]. Therefore, the data can be transferred over different networks without the need for human intervention. In the real environment of IoT applications, the smart city and home are the most popular fields. These applications mostly consist of three layers, which comprise; perception, the network, and the application [34]. Network and application layers are implemented in high-power devices that will keep data secure, while the perception layer is implemented in a low-power WSN. The WSN consists of multiple sensor nodes, which are communicating with each other by using different radio frequencies that are capable of performing various tasks of sensing, surveillance, measuring, and tracking [35]. These wireless nodes are resource-constrained devices that are characterized by their low processing power, narrow bandwidth, limited battery life, and restricted memory capacity [9]. The communication between WSN layers is depicted in Figure 2.

Based on Figure 2, wireless sensor networks are responsible for plotting the network topology and updating the routing table in the perception layer using different protocols to maintain the network infrastructure [6]. Then, the WSN starts collecting data from different locations and forwards it to the network layer (edge router). The WSN nodes are the basic building block of this layer and share some characteristics that distinguish them from other wireless networks [36]. Among these characteristics are the following:

- Independent nodes without a central control
- Stationary or mobile WSN nodes
- The transmission range of WSN nodes is also limited
- The WSN network topology is constantly changing
- Multiple hop connections
- Limited bandwidth

The WSN nodes, on the other hand, can generally operate in untrusted environments that are not regularly monitored. It is because of this vulnerability that valuable data can be easily leaked to uninvited parties, posing major security and privacy issues [37].

**Figure 3:**
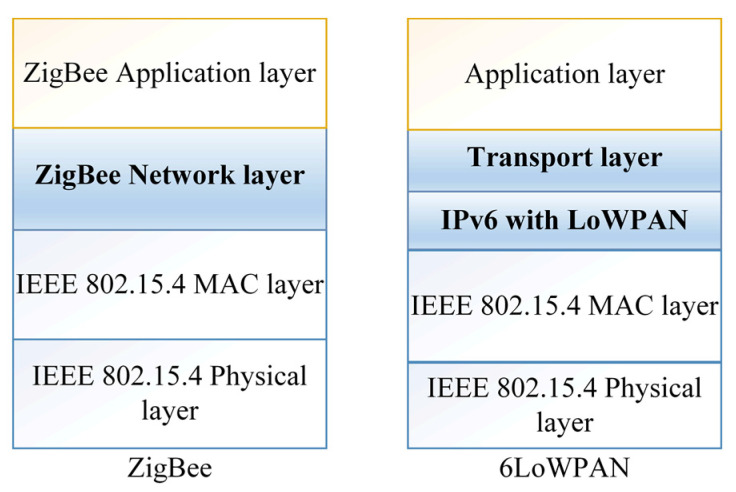
WSN co-management protocols.

The ZigBee and 6LoWPAN are two common protocols that support management in WSNs in the perception layer [3]. Moreover, they can adapt to various other network media, such as low-power Wi-Fi [38], Bluetooth [39], and sub-1 GHz radio frequency [6]. In addition, ZigBee and 6LoWPAN were compared in [40], where 6LoWPAN provides IP capabilities for WPAN networks while ZigBee offers multiple nodes that operate at low power and cost. Moreover, ZigBee can be used in home area networks and for smart metering, as well as other devices that can be intelligently monitored from a distance using this technology. ZigBee has a reliable security system and uses strong encryption technology to secure its data. Furthermore, due to channel collision avoidance, its network technology is superior to other systems. 6LoWPAN, on the other hand, is suitable for low-power IP-based systems, such as sensors and controllers. The main features in the infrastructure of these technologies are summarized in Figure 3.

However, in both protocols’ perception layers, WSN nodes are limited by computing power and energy [41]. Since the WSN is built to work in a variety of locations, it can be difficult to offer a charger in some of those locations. To circumvent this limitation, either the battery’s capacity should be raised or the security requirements should be dropped [42]. Nodes can also be charged using renewable energy, such as light, wind, and heat [43]. However, these solutions appear to be out of reach due to the size of the WSN and the requirement for additional hardware. On the other hand, decreasing security requirements allows for data breaches [44].

### 2.2. WSN Applications

**Figure 4:**
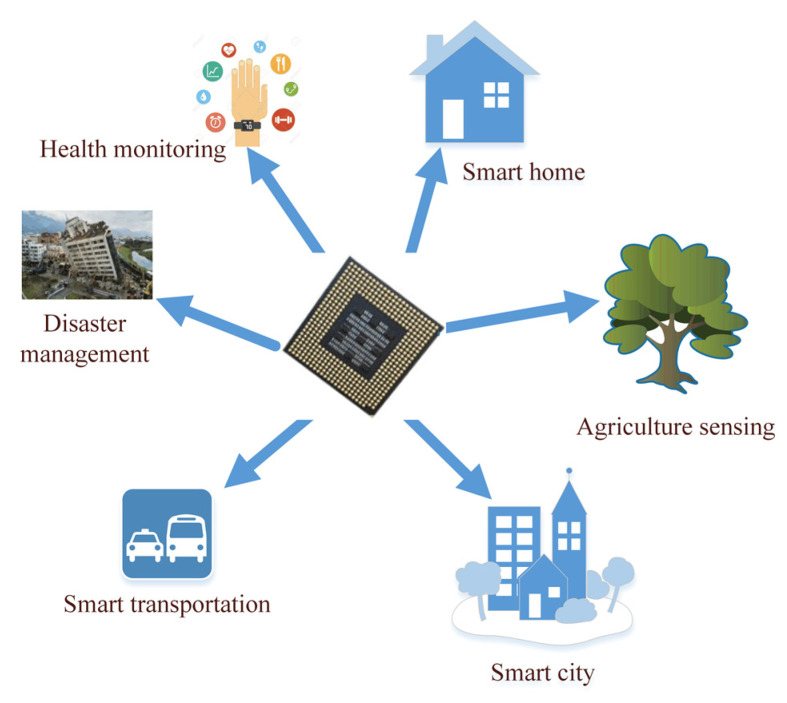
WSNs applications.

WSNs are used in many application areas, such as the military [45], healthcare monitoring [46], industrial automation [47], and smart homes [48], among others [49]. More than 50 companies have attempted to standardize a protocol running over Layer 6LoWPAN called “Thread” (https://www.threadgroup.org/, accessed on 16 May 2022) that perfectly connects and controls smart home devices [50]. Most of these types of WSNs applications [51] are shown in Figure 4.

However, due to the nature of broadcasting and wireless network vulnerabilities, attackers can quickly inject, intercept, reroute and change overhead connections [52]. This can be risky, especially when networking is used for healthcare applications [9], military applications [53,54], or the detection of human targets [55]. Any security breach can lead to dire consequences. Therefore, WSNs can be of great interest in the civilian sector when used in healthcare. However, these networks containing sensitive data need adequate protection from all kinds of potential security threats and attacks [53]. In addition to the availability of this data, the continuity of its flow is also one of the parts that must be preserved.

### 2.3. Security in WSN

A great deal of research has addressed security concerns in WSN management protocols through the Triangle defined by Confidentiality, Integration, and Authentication (CIA). What is meant by this triangle are the three axes that must be achieved in any network for it to be called secure. Confidentiality is maintaining the privacy of important data transferred between WSN nodes. In general, before sending the packet from the sending node, important segments of the packet are encrypted, and then, at the node that received the packet, the segments are decrypted [11]. In the condition of integrity, the network must be prepared to ensure that attackers cannot alter the messages sent. Attackers can create interference beams to modify their poles. In addition, before forwarding, a malicious routing node can change important data in packets. The last condition of achieving the security triangle is availability. Availability is the availability of the WSN Services at any time required. In any case, attackers can activate attacks that reduce network performance or destroy the entire network. The most harmful risk to network availability is Denial of Service (DoS) [56]; It happens in situations where attackers, by sending wireless interference, disrupting network protocols, or exhausting WSN nodes in various ways, make the network unable to set up services. This type of attack will be discussed below.

A common protocol for the transport layer in 6LoWPAN is the User Datagram Protocol (UDP), which can be overlaid with the Datagram Transport Layer Security (DTLS) protocol to ensure data security [57]. Meanwhile, TLS is operated via the Transmission Control Protocol (TCP), and the AES-128 algorithm is used for link-layer authentication and encryption [2]. However, the TLS/DTLS implementation requires additional hardware encryption hardware to maintain the use of advanced encryption operations [58]. In addition, it is difficult to integrate Internet Protocol security (IPSec) commonly used at the network layer and Transport Layer Security (TLS) into the applications of those networks because these protocols have significant overhead costs and consume significant resources [59]. Likewise, these techniques cannot fully provide the Security Triangle (CIA), since the WSN devices use wireless communication within the range of public communication channels [60]. Therefore, there must be cooperation among a set of protocols so that these types of networks can work effectively in their environments and counteract any malicious attacks. In the domain of WSN security, malicious attacks are divided into groups [44,61], and each group has an impact on sensor nodes according to the level it belongs to. The distribution of these groups at the levels of the WSN model is shown in Figure 4.

**Figure 5:**
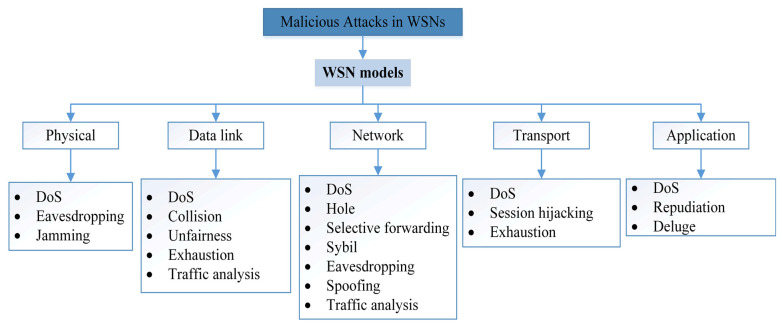
Malicious attacks classifications.

Based on Figure 5, there are different malicious attacks in each different layer, while the DoS attack shares all layers. The DoS, Jamming, Exhaustion, and Collision disrupt network connectivity and availability. Whereas Sybil, Hole, Spoofing, Session hijacking, Eavesdropping, Man in the Middle, and Selective forwarding all threaten confidentiality and integrity [62]. In addition, these attacks that hit connectivity and availability are categorized as active while others can occur in both active and passive states.

However, each of these layers has distinct tasks regarding reliability in data management and transmission between network nodes. The physical layer increase’s reliability by reducing the effect of path loss and shadowing. At the data link layer, the communication between WSN nodes must be interoperable through error recognition and multiplexing [21]. Moreover, the network layer will provide the best route for transmitting data to the edge router. However, in WSNs, each WSN node acts as a router, and the security related to this layer is responsible for securing this path from attacks. In addition, the transport layer is responsible for transmitting data to external networks, and the application layer is responsible for managing, collecting, and processing data to obtain trustworthy results [32].

Furthermore, authentication represents another important issue in the WSN security domain. For instance, the authentication method aims to protect the WSN network from being exploited by illegal WSN nodes. Moreover, different encryption and decryption methods are used in the security domain, while the limitations of WSNs lead to searching through various security technologies [63], in addition to these basic requirements in securing the security of wireless networks and who deals with them. There is also a need to track the actions of connected WSN devices to provide feedback on the events of a breach. Therefore, network security requires what is called non-repudiation, to prove actions for each WSN device [63]. Furthermore, unauthorized access to the network is faster in a WSN environment than in a wired connection, and physical entry is easy due to the hostile environment. If such user authentication is allowed, not only will the network efficiency be affected, but the data security may also be compromised. To avoid this, access control and parsing are essential, which can be provided by a variety of access control policies and encryption methods [59].

#### Attacks on WSNs

As shown in Figure 5, various types of malicious WSN attacks cause not only security issues but also other power and CPU issues. Therefore, these types of networks need to focus more on finding realistic and viable solutions than regular types of networks. In detail, we discuss the effect of each type of attack on WSNs.

1. Eavesdropping

Because of the security-related constraints of WSNs (e.g., hostile environment, dynamic nodes, and untrustworthy communication), eavesdropping is a process of acquiring information exchanged between nodes by hackers, which enhances the influence of radio fading and frequency transmission or scattering [64].

2. Jamming

This type of attack is considered one of the most dangerous types for private wireless networks. Despite its risks, security measures are ignored against it, which can cause serious problems after the implementation of wireless networks. The foremost outcome of jamming is that it impedes user service or availability due to radio frequency interference [65].

3. Collision

Since the sensors are located in different environments, this attack could be caused by malicious node replacement corruption. By presenting a brief noise packet, malicious nodes can cause collisions with surrounding broadcasts because they do not adhere to the Intermediate Access Control Protocol. Although this attack does not consume much energy from the attacker, it can lead to major network outages [66]. Moreover, due to the characteristics of wireless communication, it is difficult to determine the origin node.

4. Unfairness

This type of attack prevents authorized users from accessing network resources and exploits contract connection period settings to bypass the submission deadline [67]. Repeated collision attacks or the random exploitation of the cooperative media access control layer priority methods are examples of this type of attack.

5. Exhaustion

This type of attack recurs collision attacks until the total energy of the WSN nodes is exhausted [66]. In other words, resource depletion attacks deplete node energy by creating routing loops and path lengthening during packet transfers.

6. Traffic monitoring

In WSNs, traffic analysis is a tool for deducing patterns of communication among nodes. The analysis uses data gathered by listening in on node-to-node communication [68]. This attack specifically targets nodes that store confidential data and have the position information of the access point or sink node. As a result, if the attack is successful, a variety of knowledge is disclosed. This has the potential to be deadly to the system.

7. Hole attack

The black hole or sink attacks are network layer exploits that occur during message routing. Cluster heads are the target of this destructive bombardment. A hostile node can be chosen as the cluster head in this attack, and this node will now erase all transactional processes from its member nodes. It can potentially result in a sinkhole [69].

8. Selective forwarding

It is hard to detect a selective redirection threat, particularly when hacked nodes deliberately discard packets. Hackers can use selective redirection to establish route discovery that attracts or deletes network activity. They can also increase or decrease the range of primary routers, send bogus signals, and ignore crucial messages [70].

9. Sybil

The Sybil attack imitates the existence of a sensor node by creating several node IDs from a single current node. It also leads to system failure as a result of resource allocation issues and other issues. It has a huge effect on technologies, such as shared computing, structure management, and server protocols, which all offer load balance [31].

10. Spoofing

This attack specifically affects routing data transferred between nodes, and it can result in routing loops, root path expansions and compression, network traceability to or from selected nodes, network segmentation, bogus error messages, and elevated end-to-end latency [63].

11. Session hijacking

Another type of man-in-the-middle attack is a cookie side takeover, which gives the attacker full access to the application account. When you log in to an online account, such as Facebook or Twitter, the app sends you a ‘session cookie’, which is a piece of information that identifies the user to the server and gives them access to their account. The server will allow the user to use the app as long as their device keeps the session token.

12. Repudiation

Repudiation attacks occur when an application or system fails to implement controls to correctly monitor and log users’ activities, allowing hostile tampering or forgery of additional steps to occur. This exploit can be used to alter the data authoring of harmful user operations to log incorrect data to log files. In a similar way to spoofing electronic mail, its use can be expanded to general data processing in the name of others. If this attack succeeds, the information contained in log files may be deemed inaccurate or deceptive [63].

13. Deluge

Also known as a reprogramming assault, it is an attempt to reconfigure distributed nodes. If the assault is successful, the attacker will be able to seize control of a large portion of the network. The majority of the sensors were put in a hostile area and controlled remotely over a wireless network, which made this assault successful. It may be possible to prevent this through strong authentication.

14. DoS

This type of attack was repeated in all layers of the WSN, which means that it applies to any layer. DoS attack seeks to shut down a system or network, making it unreachable to the intended audience. DoS attacks work by flooding the victim with traffic or providing information that causes the victim to fail. A DoS attack deprives real users (workers, members, or policyholders) of the services or assets they intended to use [57].

Therefore, these attacks can affect the security infrastructure of any organization as illustrated in Table 1. Moreover, Table 2 shows the security infrastructure of WSN networks, and the protection techniques for each baseline.

In any case, malicious attack techniques change and evolve with the development of network protection software. Therefore, to be able to maintain the security of this type of wireless network, we must use the skills of self-development of sensors. The best option for their self-learning ability is to use machine learning techniques. Using these technologies, these devices can detect malicious cookies that are of a new type and not included in the current database list. The use of machine learning in WSN security is discussed in the next subsection.

### 2.4. Why Is Machine Learning Needed in WSN Security?

In malevolent circumstances, certain WSNs interact with security-sensitive information in an unsupervised manner. It is critical to use security measures for WSNs in such scenarios. Data confidentiality, data authentication, data integrity, and data freshness can all benefit from the security procedures. Traditional network security solutions, such as user authorization, are not suitable for these applications due to the WSNs’ limited resources and processing capabilities [71]. Therefore, for example, the authors in [72] designed an access gateway by using ML classification algorithms, such as Random Forest, k-NN, and Naive Bayes to assess IoT malware network activities. The k-Nearest Neighbor (k-NN) method showed the highest accuracy, according to the outcomes of performance assessment with those kinds of techniques. Moreover, the authors in [73] presented a privacy-preserving Support Vector Machine (SVM) training method for IoT data that requires only two transactions in one iteration and does not require the use of a reliable third party. When compared to conventional SVM, this technique greatly reduced computational complexity.

Therefore, ML technology provides a good model for reducing the cost of some areas of security. Anomaly detection, for example, provided excellent results against all types of malicious activity, and in the process of packet analysis [64,66,74], tracking and protection against DoS [20,21,67,75,76,77,78]. The processes of raising the availability of networks, error detection [23,24,25] and traffic congestion [17,18,19] are also based on the ML approach. In addition to the authentication operations of the physical layer, it can be a good solution [20,21,22]. Therefore, the application of ML techniques in WSNs aims to solve many of these problems and provide tremendous advantages in terms of flexibility and accuracy.

## 3. Machine Learning Techniques

**Figure 6:**
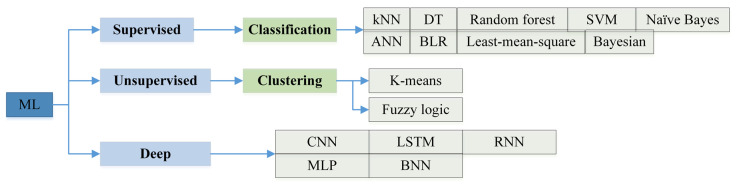
Classification of ML algorithms used in the security of WSNs.

In this section, an introduction to the types of machine learning algorithms that were used in WSNs security is provided. These algorithms are divided into several categories, including supervised, unsupervised, reinforcement learning, and deep learning. Therefore, in this section, we review the classifications of ML algorithms that have been used in the reviewed studies in Section 5. This is because many surveys specialize in the field of machine learning comprehensively and operationally. The classification of used ML algorithms is illustrated in Figure 6.

A brief explanation of how ML algorithms work is provided here, whereas many research papers published in this field, such as [27,52,79,80] discussed how these algorithms work in detail.

### 3.1. Supervised Learning

**Figure 7:**
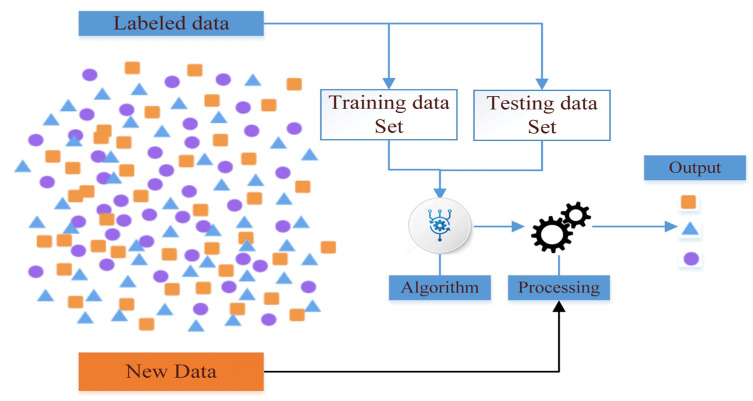
Supervised learning processes.

Supervised learning is a ML task that infers functions from labeled training data sets. Training data consists of a set of training examples, each example is a pair consisting of an input object (usually a vector) and the desired output value (also called a supervised indicator). The supervised learning algorithm analyzes the training data and produces an inferred function that can be used to map new examples. An optimal solution to the algorithm will allow to correctly identify the category label when the label is not visible. Therefore, samples with certain properties known as a training set, are used to create a mathematical model (such as the distinctive model in pattern recognition, and the weight model in the artificial neural network method), and then the adopted model is used to predict the unknown samples [80]. Figure 7 shows the training processes for supervised learning on datasets in a simplified manner [81].

However, supervised learning offers a good ability to predict future samples with high performance and accuracy, but it needs high computational time and CPU through the training process and these limitations make the training process difficult to apply to applications that need real-time outputs. The algorithms that have used this are:

#### 3.1.1. k-Nearest Neighbor

In theory, the k-Nearest Neighbor (kNN) classification technique is one of the simplest ML methods available. In this strategy, if the nearest neighbors of a sample in the feature vector, the most comparable k samples, belong to a certain class, then the same sample also belongs. The K-nearest method for the training data set and, as an example, a new entry is given. The algorithm finds the most closely related k instances and classifies the inputs as belonging to this class [82].

#### 3.1.2. Decision Tree

The Decision Tree (DT) method is used to start from observations about a particular item to the value it represents in the tree leaves. It is a predictive modeling method that is used in statistics, data mining, and mapping learning. Furthermore, variables contain a set of values in decision trees called classification trees where the leaves are represented in the form of a tree to do what they represent in the branches. Decision trees that target numeric variables with real numbers are called regression trees (relative to linear regression). Decision-making arises from ongoing decision-making in data mining operations related to data management (but the output of the classification tree is an input into the decision-making process) [83].

The general motivation for using a decision tree is to create a training model that can be used to predict the category or value of target variables by learning decision rules inferred from past data (training data). Therefore, the level of understanding of the decision tree algorithm is very easy compared to other classification algorithms [84].

#### 3.1.3. Random Forest

Using the Random Forest approach, a data set’s predicted accuracy is improved by combining several decision trees on distinct subsets. To forecast the ultimate output, the random forest uses predictions from each tree, rather than a single decision tree [85]. As there are more trees in a forest, accuracy increases, and overfitting is avoided.

#### 3.1.4. Supportive Vector Machine

**Figure 8:**
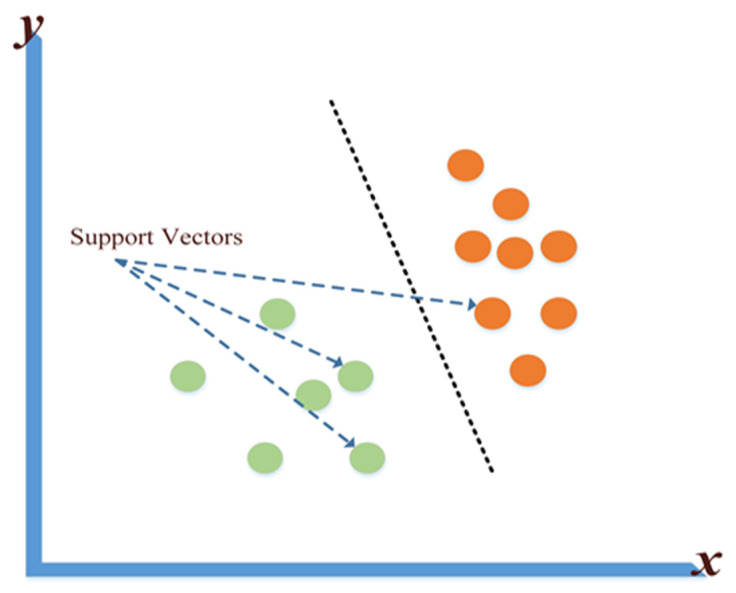
The SVM method.

The Supportive Vector Machine (SVM) algorithm is a supervised machine learning algorithm that can be used in Classification or Regression problems. Its main use is in classification by giving each data element a point that is plotted in an n-dimensional space, with the value of each attribute being a given coordinate. To classify two classes, the hyper line that separates them is determined. Figure 8 shows what was indicated [86].

#### 3.1.5. Naïve Bayes

The naïve Bayes is a classification method based on Bayes’ theorem and independent assumption of characteristic conditions. For a given training data set, we first find out the combined input/output probability distribution based on the independent hypothesis of feature conditions. Then, based on this model, for the given input x, we use Bayes’ theorem to find the output with the greatest subsequent probability y [87]. In more detail, this classification system should first be trained on a set of learning data that shows the expected class according to the entries. The algorithm constructs its classification rules of this data set during the learning phase, then applies them to the classification of a prediction data set a second time. The supervised nature of the naïve Bayes classifier assumes that the learning dataset’s classes are known and provided [88].

#### 3.1.6. Artificial Neural Network

An Artificial Neural Network (ANN) is a technique for classifying data based on a human neuron model. An artificial neural network (ANN) is made up of a large number of neurons (functioning units) that digest data and deliver correct outputs. Layers are commonly used in ANN, with nodes connecting the layers and each node having an active duty [89]. Each ANN consists of three layers: an input layer, one or more hidden layers(s), and one or more output layers. ANN is highly good at classifying complex and non-linear data sets, and unlike other classification algorithms, there are no input restrictions.

#### 3.1.7. Logistic Regression

Logistic Regression (LR) is a statistical technique that seeks to construct a model that allows a set of quantitative or qualitative descriptive factors to estimate or describe the values taken by a qualitative attribute value (most typically binary). When the dependent variable is dichotomous, LR is also described as a technique for matching a regression model to the data [90]. This method is used to see if the independent variables might forecast a dichotomous dependent variable in research.

#### 3.1.8. Least-Mean-Square

The Least Mean Square (LMS) method is a type of ML filter that employs stochastic gradient descent in complex terms. Gradient descent is used to continually update the filter weights to estimate the output. Moreover, LMS requires specific learning curves in ML theory and practice thanks to the principle of algorithm convergence. These ideas focus on optimizing ML models, fitting inputs to outputs, improving training and testing methods, and generally achieving “convergence”, in which the repetitive learning process merges into a cohesive result rather than deviating [91].

#### 3.1.9. Bayesian

Bayesian is a supervised ML method [50] that is based on statistical learning methods. By learning conditional independence using different statistical methods, Bayesian learning discovers correlations between datasets. The probability p ($|X_1_, X_2_, X_3_, …, X_n_) to be maximized is given by a collection of inputs X_1_, X_2_, X_3_, …, X_n_. Furthermore, Distinct probability functions for different factors of class nodes can be used with Bayesian learning [90].

### 3.2. Unsupervised Learning

Unsupervised learning is also one of the major branches of machine learning and artificial neural networks. Machine learning algorithms are trained by distinguishing patterns of data without knowing the output of the data (unlabeled). One of the primary applications of unsupervised learning is estimating data density to find commonalities between items and arrange them statistically. Moreover, if compared with supervised learning, it can be said that the difference between them is that the first (supervised) works to infer the initial distribution of data, while the other works to infer a knowledge-conditioned graphical distribution from additional factors [92].

However, unsupervised learning provides less complexity and is faster to implement, but the accuracy of the output prediction is less accurate.

The algorithms that have been used of such a type are:

#### 3.2.1. K-Means

The k-means clustering algorithm is a ML algorithm that groups points close to each other into clusters. In this algorithm, there is no learning model construction because the new point in any group is challenged based on its distance from all groups (often its distance from the group center or its arithmetic mean) and placed within the group to which it is closest. For example, dividing a group of points into a line of three groups. To determine how close a point is to a particular group, it will use a measure of how far it is from the group (for example, the distance between two points) [93].

#### 3.2.2. Fuzzy Logic

The goal of fuzzy logic is to insert values (degrees) between real numbers (elements), with the fuzzy group assigning a degree of membership to the universe’s elements, which is commonly a real number with a period of [0, 1]. Propositions are given degrees of truth, which gives rise to fuzzy logic. The current standard of truth values (scores) is [0, 1], with 0 denoting “completely false”, 1 denoting “absolutely true”, and the remaining numbers denoting partial truth, i.e., intermediate degrees of truth [94]. Therefore, Fuzzy logic is used to resolve issues involving imprecision, ambiguity, estimates, ambiguity, qualitative chaos, and partial truth.

### 3.3. Deep Learning

**Figure 9:**
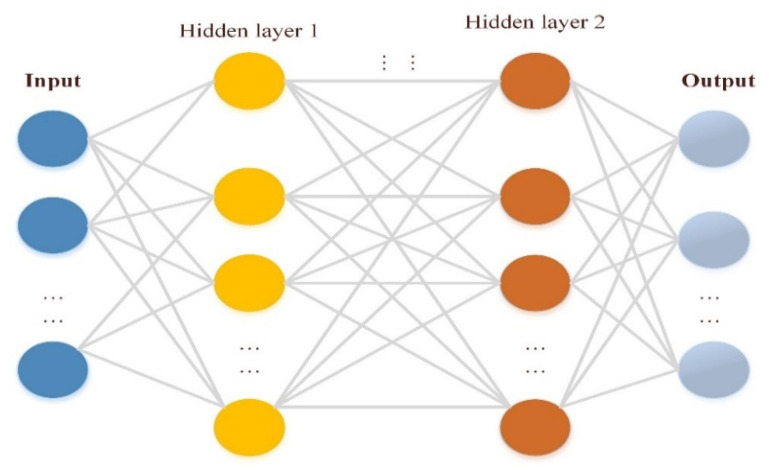
Deep learning technique.

Deep learning is a subtype of ANN classification technology, in which ways to represent data learning with multi-layer representations are known as deep learning techniques (between the input layer and the output layer). The deep learning technique is illustrated in Figure 9. It is made up of basic non-linear modules that convert the description from a lower to a higher layer to reach the optimal result [95]. Deep learning has several advantages, including the ability to extract high-level characteristics of data, the ability to function with or without labels, and the ability to be trained to achieve various goals.

The algorithms that have been used of such type are:

#### 3.3.1. Convolutional Neural Networks

**Figure 10:**
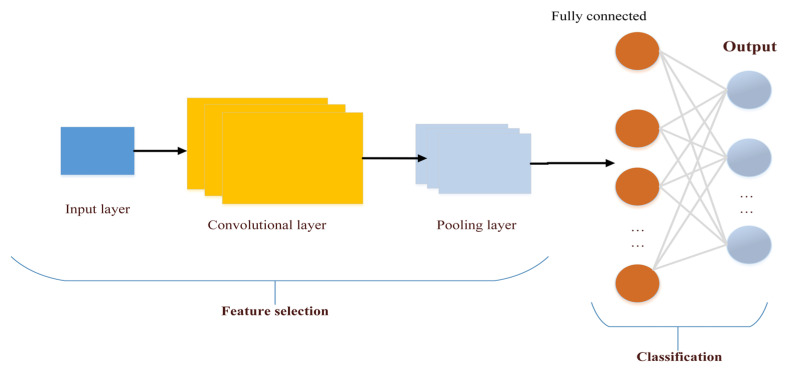
CNN architecture.

Convolutional Neural Networks (CNNs) are deep learning systems that are comparable to a multi-layer Perceptron at their foundation but differ in what they learn, how they are built, and what their purpose is. Moreover, CNNs are often used in various applications through data analysis. In the first stage, the process of identifying and extracting feature selections is performed, then the classification process is performed [96]. It is characterized by having one or more hidden layers which extract the attributes in images or videos, and a fully linked layer to produce the desired output [97]. The algorithm consists of different layers starting from the convolutional layer, the activating function, the padding layer, the pooling layer, and ending with the fully connected layer. Moreover, each layer is oriented in a different shape or pattern. The first layer serves to define straight lines and the other works to define circles, and so on, the layers continue until they can finally determine what they were designed for. Figure 10 shows the architecture mechanism of the CNN algorithm [98].

#### 3.3.2. Recurrent Neural Networks

Recurring Neural Networks (RNN) or Recurrent Neural Networks specialize in understanding and manipulating sequences of different types, therefore, they are used in many fields, such as machine translation, manipulation of human genetic code, and many other fields. Moreover, RNNs have a “memory” that allows them to take information from previous input data to influence current input and output. Unlike other deep neural networks that assume that the inputs are independent of the outputs, the output in RNNs depends on the previous elements in the data sequence [99]. The RNN, on the other hand, has short-term memory difficulty. It will have difficulty transporting data from earlier generation steps to later ones if the sequence is long enough.

#### 3.3.3. Long-Term Short Memory

Long-term memory (LSTM) is a special type of RNN that can learn dependent long-term information. LSTMs were specifically developed to prevent the long-term dependency problem. Gates is the basic principle of LSTMs. As a kind of information superhighway, the state of the cell acts as a channel for transmitting relative information along the chain. you can think of it as the “memory” of the network. As a result of the state of the cell, the sequencing process can be affected. Thus, even knowledge from previous age steps can work its way into later time steps, reducing the effect of short memory on learning and recall of new knowledge. Information is added or pulled from the cell as it travels [100]. However, cell state knowledge is controlled by distinct neural networks.

#### 3.3.4. Multi-Layer Perceptron

Multi-Layer Perceptron (MLP) is a form of feedforward artificial neural network. A linear perceptron may identify data that are not linearly separable because of its numerous layers and non-linear activation. There are at least three levels of nodes in the MLP: an input layer, a hidden layer, and an output layer. It is a neuron that uses a nonlinear activation function except for the input nodes. Moreover, MLP uses a technique called backpropagation for training, which is supervised learning [101].

#### 3.3.5. Backpropagation Neural Networks

Backpropagation, or backward error propagation, is a popular tool for calculating derivatives in deep feedforward neural networks. It is used in several supervised learning algorithms for training feedforward neural networks, including stochastic gradient descent. While training a neural network using gradient descent, it will calculate a loss rate that measures the difference between your predictions and the real labels. Backpropagation allows us to determine the gradient of the loss function concerning each of the network’s weights. Thus, the loss function can be reduced across numerous model training sessions by updating each weight separately [102].

Consequently, Backpropagation is a reverse process of gradient calculating as it moves backward through the feed-forward network from the last layer to the first layer. The chain rule is used in calculus to group the gradations of all subsequent levels to determine the gradient in a particular layer.

## 4. WSN Security Challenges

### 4.1. Challenges of WSN Security

Wireless sensor network technology is an efficient arrangement of data collection and real-time data transmission through the perception layer. However, this layer leads to limitations on the entire network infrastructure, particularly its reliance on public wireless channels.

**Figure 11:**
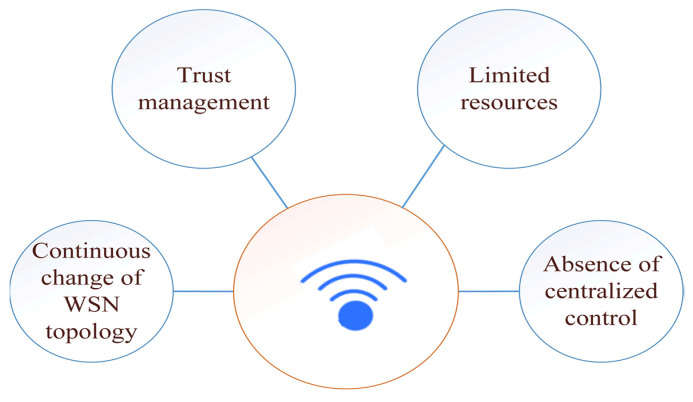
WSN’s main security challenges.

Based on the characteristics of the WSN nodes discussed above, there are many challenges, especially in the field of security. Moreover, since the implementation of the previously mentioned network security requirements on wireless sensor networks is a challenge, security and data privacy in wireless sensor networks is another challenge. Figure 11 presents the main challenges facing WSNs security, and we discuss these factors as follows:

#### 4.1.1. Absence of Centralized Control

Due to the absence of centralization in the perception layer, authentication operations, for example, will take place individually between adjacent WSN nodes. Therefore, the use of protocols based on dividing the WSN nodes into clusters and sharing the same authentication by adjacent WSN nodes is an acceptable solution [9].

#### 4.1.2. WSNs Topology Changes

Due to WSN node movement, environmental modifications, and the addition of new WSN nodes or the loss of current WSN nodes, the WSN network topology is always shifting [103]. As a result, protocols that deal with those topological changes, such as routing and authentication protocols that allow multi-hop communication, are required. For example, the signal is shared between the transmitting WSN node and all neighboring (receiving) WSN nodes in the transmitter’s transmission range, each time the network infrastructure is renewed.

#### 4.1.3. Scalable Trust Management

In WSNs, trust management is the difficulty of identifying legitimate nodes from illegitimate nodes. The occurrence of a breach and the need to withdraw trust when it is detected, power limitations, the number of nodes to consider, and the difficulty of rebuilding trust when breaches occur are all unique challenges to trust in sensor network management [104]. Furthermore, due to the performance/energy limits of several of the WSN nodes, it may not be able to accomplish complex key generation methods or pairs between them. Even if this is possible once, it may not be practicable to do it regularly. Because it is assumed that a physical breach of certain WSN nodes (and hence their shared keys) is inevitable, limits on the number of nodes sharing keys should be imposed to reduce the impact of an attack. This process can be done by developing lightweight key management approaches [105].

In addition, each node of these WSN nodes must have a minimum level of trust from neighboring nodes to be able to send and receive data from and to them. Suspicious packets must be detected during the construction of the network infrastructure or during the process of sending and receiving data at each node. Each node must have the Introduction Deduction model to be able to protect itself and its peers, and this is done using lightweight self-development skills.

#### 4.1.4. Limited Resources

Limited resources are another challenge caused by the limited work of the WSN node in collecting the data for which this sensor is intended. Therefore, it is very important to reduce the cost of these devices to suit the needs of users, and this comes with challenges in the security of these devices as a result of their weakness in securing the minimum level of protection for themselves [106].

However, security management in WSNs should not bear a lot of connections, computation, and storage, and it should be compatible with other network management functions.

### 4.2. Challenges of Using ML Algorithms in WSN Security

Despite the importance of machine learning techniques in developing the skills of WSN nodes to detect vulnerabilities or malicious attacks. There are many challenges for this type of wireless network due to its limited energy and CPU capabilities [27]. These challenges are illustrated in Table 3.

1. Machine learning algorithms, which include learning from historical data, cannot make accurate real-time predictions. The amount of additional data determines the efficiency of the algorithm. When the amount of data is huge, the cost of energy required to process it is equally large. In other words, there is a trade-off between the power limitations of the WSN and the higher computing burden of the ML algorithm. ML algorithms must be implemented centrally to avoid this trade-off. Therefore, these algorithms pose a risk [27] for wireless sensor network environments.
2. Machine learning techniques cannot be applied to all WSN’s security requirements. Sometimes it is difficult to apply them to some security domains, such as authentication and integrity [107]. Providing such operations between WSN nodes requires a high CPU and power. This can be represented by authentication between the vehicle and the driver, for example, but it is difficult to represent between one WSN node and another [108]. On the other hand, some studies have used ML algorithms for authentication through physical channel exploits [109]. These ML techniques are discussed in Section 5.2.
3. Most machine learning algorithms have a margin of error, even if this margin is small, it is there. Therefore, in secret data, its confidentiality should be close to perfect [110]. The authors worked in [111] by providing a Mathematical Encryption Standard (MES) to increase case-based risk monitoring of confidential healthcare data using ML technology. Decision-making regarding the risk control strategy in MES was enhanced based on a fuzzy inference system integrated with neural networks. Analysis of the results shows that the MES error rate is less than 0.05 and the accuracy rate is 97%, which indicates their desire to increase security risks. Despite the improvements made by the authors, there is still an error rate, even if it is close to zero.

## 5. Applications of ML to Secure WSN Networks

In this section, we discuss the applications of ML algorithms in the security of WSNs. Based on what was previously discussed about the security requirements in the WSNs, we review the role of ML algorithms to cover these requirements.

Most security applications of ML have been used in intrusion detection technology to help understand the movement of packets in the network [112]. Part of these ML algorithms helps provide network availability by reducing DDoS and DoS attacks. Others help analyze the behavior of viruses and reduce their data integrity risks, such as ransomware attacks [113]. Furthermore, some ML technologies contribute to helping prevent authentication attacks between WSN nodes. All these subsections will be presented in detail in the following sections.

### 5.1. Availability

Availability is one of the main requirements for security in networks. Thus, under the name of availability, many intentional or unintended attacks, such as DoS, equipment damage, or power reach the bottom line in WSN devices. Intrusion detection, error detection, and congestion control, for example, are ways to increase the availability of networks.

#### 5.1.1. Intrusion Detection

In general, the intrusion detection system’s major tasks are to scan networks and hosts, evaluate network activity, produce alarms, and react to suspicious activity. Intrusion detection systems are often deployed near secured network devices since they monitor linked hosts and connections (e.g., the switches) [15]. In WSNs, all WSN nodes act as hosts and network devices (router and switches), therefore, each node must perform the same intrusion detection process on its own. Detection is of two types, either Signature-based or anomaly-based, preferably based on anomalies in terms of learning skills to WSN nodes. However, the problem remains, as we explain in the WSN Challenges subsection, which is the ML training process. Therefore, many studies in this part have attempted to improve the machine learning training process in the wireless sensor network by reducing training time, relying on a small data set, and improving accuracy. Table 4 summarizes the ML algorithms in the application of intrusion detection.

Authors in [114] proposed a new model to improve DoS detection and save power consumption in WSNs. The authors also proposed a new cluster model in the LEACH protocol to distribute forwarding messages between WSN nodes. After that, they used feature selection, along with a classifier algorithm to improve DDoS Attack detection. Feature selection is another technique used to reduce features in a dataset by selecting the most important features for the training process and excluding the rest. In addition, the authors attempted to determine the power consumption of their proposed method on WSN and found that it increases power consumption by 5%. The authors also found that one of the best machine learning techniques for protecting wireless sensor networks from DoS is the decision tree with a 100% accurate result. Moreover, the authors in [115] analyzed the effect of different ML algorithms for DoS detection in WSNs. They chose ML algorithms of different types (statistical, logical, instance, and deep learning) and applied them to different dataset sizes to study the effect of data volume on the training process in ML algorithms. Moreover, they studied the lightweight ML algorithms in WSN nodes. From the results, it was found that the best dataset sizes are between 3000 and 6000 records, provided that the ratios between attacked and non-attacked records are 1-1. The results also showed that the best classifiers are those that also belong to the logic-based (decision tree), which is the G-boost. Furthermore, the best performing algorithm for DoS detections increased the power consumption of the network by 32%. Moreover, in the same context of analyzing the traditional machine learning algorithms and deep learning on the traffic packets of wireless sensor networks. The authors in [86] have proven that simple models, such as (LR, DT, and SVM) are ideal for the real application of intrusion detection from deep learning methods.

Another approach has been suggested for online DoS detection using statistical analysis in [116]. The authors used binary logistic regression in the forward-selective and black-hole attacks. First, a run-time monitor tool was used to aggregate the local WSN node activity, whether they were benign or malicious packets, and then binary logistic regression was applied to find out the detection accuracy. Then they installed the output of the algorithm (logistic regression) on the WSN network to measure the activity of nodes in detecting attacks. The accuracy of their suggestion was between 96–100%. Another rule-based ML approach was proposed in [117]. The authors created hybrid ways that combine fuzzy logic and other techniques along with a rule-based approach to deal with ambiguities, inaccuracy, and vagueness. Then they evaluated the reliability of those traits.

In [75], the authors proposed a new model to improve network lifetime combined with intrusion detection efficiency. To optimize the power consumption of a WSN node, the authors proposed an adaptive chicken swarm optimization algorithm, and for intrusion detection, the authors used two levels of the SVM method. At the first level, the SVM will be used to detect the malicious node, and at the second level, the SVM will be used to inspect packets. However, the paper discussed the issue of improving WSN lifetime, but the results do not contain any explanations for how much energy the proposed method has saved. In addition, the authors in [30] used a deep neural network (DNN) to develop a flexible intrusion detection method. The results also showed an improvement in the accuracy of the results for different types of network traffic. However, the paper also discussed the performance accuracy of the proposed method without mentioning the cost of the proposal in power and CPU. Furthermore, the authors in [78] proposed a lightweight intrusion detection technique for WSN networks by combining particle swarm optimization (PSO) and the backpropagation neural network (BNN). In [118], the authors proposed a hybrid feature selection method along with a two-level classifier (rotation forest and bagging) to improve the performance of intrusion detection accuracy. Additionally, SVM was used with MLP in [119] to classify traffic data and identify malicious nodes in the WSN network.

In a different direction, some authors have produced a hybrid classifier between synthetic groups of machine learning algorithms. The authors in [120] proposed a hybrid classifier that combines deep learning with traditional machine learning techniques. The proposal used a combination of the LTSM model and the Gaussian Bayes model to improve intrusion detection in WSNs. Whereas the proposal in [77] used a combination of the MLP model and the Genetic Algorithm (GA).

However, the proposed algorithms discussed earlier in intrusion detection all consume quite a bit of power. Therefore, in some other studies, Software Defined Network (SDN) technology [121] was used to transfer the training process to the console instead of the WSN node. Therefore, these ideas are rather good at reducing the effort on WSN nodes. However, these methods need to modify several protocols that occur between switches, controllers, and WSN nodes to pass the training results to the sensors on time.

Authors in [122] distributed machine learning methods for intrusion detection training in a hierarchical approach between controller and switch, where terminal nodes avoid any consequences of detection processes. In the controller, the first stage of training was carried out using a decision tree, KNN, NB, and LR, then the switches were carried out in the second stage of training. However, the study did not explain the improvements and modifications it made to the SDN protocols to enable their proposal. Additionally, in [123], the authors used KNN with the arithmetic optimization algorithm (AOA) in evolutionary computation to produce an advanced intelligence framework. Moreover, in the same context of using SDN, the authors in [124] used it to improve the detection of phishing attacks. The optimization was based on the combination of traditional methods (blacklist and whitelist) with the features extraction process, which relied on the URL and content of websites. The blacklist and whitelist are updated based on the output of features extraction of packets that come from users. The naïve Bayes classifier was used for the feature extraction process. Next, the controller updates the flow rule table and then sends it to switches to perform actions for each packet that matches those rules. If the packet does not match any value in the rule action table, the previous process will be repeated. Despite the improvements shown by their results, the proposed solution is large and complex. Similar to the same approach, the authors in [125] used machine learning based on stacking methods to detect the URL packets that are not blacklisted or whitelisted. Moreover, the authors in [126,127] used CNN along with SDN to improve the URL detection accuracy. The CNN is used in the controller to classify the URL in a signature-based database to different types of phishing attacks. Based on this classification, the coming packet inspection will either be forwarded directly to the destination or go into slow mode. In the slow mode, it will perform more inspections to update the signature-based database. However, all three did not consider feature selection in their proposals despite its economic feasibility in reducing the training and improving performance.

**Table 4:** Summary of reviewed ML algorithms in intrusion detection.

Refs.	ML Technique	Processing Cost	Advantage	Limitations
[114]	Water Cycle + DT	Low	Improved detection accuracy Reduced WSN power consumption	The analysis covered one type of WSN packet traffic
[115]	Various ML algorithms	-	Determine which types of ML algorithms are best for WSN intrusion detection Determine which data set size is best for WSN intrusion detection	The analysis covered one type of WSN packet traffic
[84]	Various ML algorithms	-	Determine which types of ML algorithms are best for WSN intrusion detection	The analysis covered one type of WSN packet traffic The analysis did not discuss the impact of intrusion detection on WSN energy consumption
[116]	BLR	low	Improved detection accuracy Calculated the intrusion detection cost power on WSN	There were not enough benchmarks studies
[117]	Fuzzy logic association rules	medium	Improved detection accuracy	There was no analysis of intrusion detection power consumption in WSN
[75]	Two levels of SVM	Medium	Improved detection accuracy Improved bandwidth	WSN power consumption was not discussed
[30]	DNN	High	Improved detection accuracy	There was no analysis of intrusion detection power consumption in WSN
[78]	PSO and BNN	High	Improved detection accuracy	There was no analysis of intrusion detection power consumption in WSN
[118]	PSO, GA, rotation forest, and bagging	High	Improved detection accuracy	There was no analysis of intrusion detection power consumption in WSN
[119]	SVM + MLP	High	Improved detection accuracy	Decreased accuracy over actual scenarios
[120]	LTSM + Gaussian Bayes	High	Improved detection accuracy Calculated the intrusion detection cost power on WSN	There were not enough benchmarks studies
[77]	MLP + GA	High	Improved detection accuracy	There was no analysis of intrusion detection power consumption in WSN
[122]	SDN + different ML algorithms	Low	Improved detection accuracy Intrusion detection time consumption	There was no analysis of intrusion detection power consumption in WSN There was no discussion about updating SDN protocols
[123]	KNN + AOA		Enhanced detection accuracy	WSN power consumption was not discussed
[124]	SDN + naïve Bayes	Low	Improved detection accuracy Intrusion detection time consumption	There was no analysis of intrusion detection power consumption in WSN
[125]	SDN + TIER-1	Low	Improved detection accuracy	There was no analysis of intrusion detection power consumption in WSN There was no discussion about updating SDN protocols
[126]	SDN + CNN	Low	Improved detection accuracy Intrusion detection time consumption	There was no analysis of intrusion detection power consumption in WSN
[127]	SDN + CNN	Low	Improve detection accuracy of intrusion detection Transferring the cost of detection from the devices to the SDN-switch	SDN-Switch Congestion Presence

#### 5.1.2. Error Detection

In error detection, machine learning algorithms provide a great example. The WSNs are also error-prone and malfunctioning as a result of their various software, hardware problems, and implementation in various domains. Because of all these difficulties, significant application detection techniques must be employed to quickly discover flaws in a WSN. The authors employed a trust mechanism decision fusion method in [23]. To boost the effectiveness of the belief function fusion approach, four categorization strategies are given which are KNN, extreme learning machine, SVM, and recurrent learning machine. However, the dynamics of specific WSN node malfunctions are not captured by this method. For the dynamic capturing of the WSN nodes during fault incidence, the authors in [128] used a hidden Markov model to determine the dynamics of transitions caused by an error, and neural networks were used to classify faults based on the state transition probability generated by the Markov model. As a result, the authors focused on error detection and classification using a combination of a hidden Markov model and various neural networks, such as learning vector quantization, probabilistic neural network, probabilistic adaptive neural network, and radial basis function.

In the classical ML algorithms, the authors in [129] used SVM classification for error detection in WSNs while the authors in [130] used the SVM regression model for the same purpose. Moreover, using recursive PCA and a multi-class SVDD classifier, the authors of [131] described an online error detection method for real-time data flows. The lightweight recursive principal component analysis method was utilized to discover the error in WSNs. The error types were identified using the SVDD classifier. The failure to detect a malfunction in the body sensor network can result in a mistaken medical diagnosis, hence it is critical to do so. In [24], a Bayesian network-based error detection method for the body sensor network was reported. The temporal and geographical correlation of body sensors were captured using a Bayesian network technique. Based on an appropriate threshold setting, sensor errors can be determined. Furthermore, in [25], the authors offered an error detection strategy for detecting problematic nodes in WSNs using their batteries and WSN node data. The fault nodes can be identified in the proposal during two-level validation. A Naive Bayesian classifier was implemented to identify the error within the sensor node during the first stage, and the error detection through the block header or the gate was assessed in the second stage. Through simulation results, this technology demonstrates a 100 percent accuracy rate. In addition, a defective node allocation and management strategy in wireless networks based on fuzzy criteria was given in [132]. The major goal of this approach was to reuse faulty WSN nodes by providing the most effective routes to the base station. It improves service reliability and network longevity. Based on the anomalous behavior of the sensors, a k-NN classifier has been used to distinguish the error WSN nodes from the normal WSN nodes. This module relies on the WSN node error rate in order to locate malfunctioning WSN nodes.

Table 5 provides a good analysis of the reviewed studies in detecting errors.

#### 5.1.3. Congestion Control

Congestion control is considered by some to be part of the quality of service, but it can also be seen as one of the tasks that contribute to network availability. Furthermore, machine learning algorithms contribute well to this area.

In WSNs, congestion occurs when a WSN node or communication channel receives more data than it can process. Buffer node bypass, transmission channel contention, multi-to-one data transmission systems, Packet collision, dynamic time shift, and transmission rate are just a few of the causes of congestion [27]. As a result of the congestion, energy consumption, packet loss, and end-to-end delay are all affected [29]. ML algorithms can help with congestion control issues by estimating network traffic and finding the optimal path.

The authors in [17] used an active queue management protocol called Random Early Detection (RED), to detect congestion and determine the potential for packet loss. This protocol tends to reduce the buffering queue and adjust the data transmission of each WSN node by integrating fuzzy logic and Proportion Integration Differentiation theory. Congestion identification, congestion reporting, and transmission rate modification are the three steps in this system. RED and fuzzy proportional integral derivative (FuzzyPID) controller approaches are used to identify congestion first. When congestion is identified, implicit congestion reporting is created. Finally, a fuzzy controller is used to manage congestion by adjusting the transmission rate.

In [133], the authors also used an active queue management protocol that uses buffer occupancy to sense congestion. It estimates the amount of packet loss based on the length of the current queue and changes the queue length accordingly. The relative integration differentiation control theory is used for the first time in WSN node queue management in their proposal. Then, using the self-learning and self-regulating capabilities of the neurons, an online weight setting is generated to configure the percentage, integral and differential parameters of the relative integration differentiation controller. Finally, to accomplish an online optimization, the control parameters of proportion, integral and differential parameters, and neuron learning rates are taken into consideration using the usual particle swarm optimization to neural relative integration differentiation technique. Furthermore, in the same context of using fuzzy logic, the authors in [18] used the fuzzy clustering technique in cluster nodes to solve the congestion control problem when the cluster node buffer is full. Two-tiered Fuzzy Logic is described in this diagram, sensor nodes attempt to estimate the load profile based on previous run loads using ARIMA technology, and Fuzzy Logic selects the closest uncongested sensor nodes from several eligible mobile sensor nodes based on it. Then the result of the first fuzzy logic is used by the second fuzzy logic to choose the appropriate nodes as cluster vertices, which reduces network power consumption.

The authors in [19] proposed a heuristic strategy based on learning Real-Time (A Star) for finding the most powerful optimal route. The author focused on altering the node degree and topology. To avoid congestion, the data flow is then balanced utilizing fuzzy logic. If there is traffic, it uses real-time learning to locate an alternate optimum path. Moreover, to reduce energy usage across the network, a rate-dependent congestion control method based on cluster routing has been developed [134]. Rate control technology reduces end-to-end lag and extends the system life over a longer period. The combination of K-mean and Greedy is the first method for searching for cluster nodes in the beginning. The rate management is then implemented with the help of the Firefly optimization approach, which is designed for high packet delivery ratios. Finally, ant colony optimization-based routing is used to send packets with the highest possible throughput. In [135], the authors proposed a fuzzy sliding manner congestion management technique for WSNs to address the congestion problem. To start, a new cross-layer congestion control model has been proposed between the transport layer and the data link layer by incorporating the signal-to-noise ratio of the wireless channel into the TCP model. Then, by integrating fuzzy control with sliding mode control, a fuzzy sliding mode controller is created, which adaptively modifies the buffer queue length in crowded nodes while drastically reducing the impact of uncertain external perturbations.

Table 6 provides a good analysis of the reviewed studies in congestion control.

### 5.2. Authentication

Authentication refers to a set of security forces that ensure data have come from the source and have not been tampered with along the way [136]. Its approach ensures that active attacks, such as DoS and spoofing are mitigated. Authentication encompasses both the network element and message features. Since both the claimant and the verifier communicate and interact without giving any critical info other than the claim of becoming a specific entity, entity authentication is achieved. While message authentication does not ensure when a message was generated, it would provide an appropriateness guarantee. In traditional networks, traditional public-key cryptography schemes and algorithms, such as RSA [137], ECC [138], Defihelman [139], and others are used in the process of authentication [140]. However, due to the wireless sensor network characteristics discussed earlier, the implementation of such mechanisms leads to power exhaustion. In addition to modern methods of authentication based on motion sensors for users (devices or humans), it can be used in many works, but it also relies on a high processor and battery capabilities. Therefore, the authentication process using a physical layer is a good option for wireless sensor network environments. Table 7 summarizes the ML algorithms in the authentication implementation.

Machine learning techniques can reduce WSN power consumption by performing physical layer authentication. Authors in [141] suggested a physical layer authentication method that uses LSTM to learn about wireless hardware flows. Their proposal exploited the temporal correlation between I (Preamble phase)/Q (Quadrature phase) of wireless signals to distinguish low-power transmitters from high-power competitors. Furthermore, compared to other ML algorithms, their results showed that deep learning algorithms have greater accuracy than regular ML algorithms. In the same context of using physical layer authentication, the authors in [21] created a deep learning-based physical layer authentication framework to improve the security of industrial WSNs. Several WSN nodes in various places of the industrial environment have been detected by application level authentication to simplify labeling the matching channel state information before transmission. Moreover, the authors in [22] used radio channel information and the ML technique to authenticate WSN nodes. The authors trace the radio channel similarity between the adjacent transmission interval over a specified period (threshold) between legitimate and illegitimate users. In addition, for the adaptive authentication in a dynamic environment, the authors in [142] trace multiple physical layer attributes based on a kernel-based ML technique. The suggested technique decreases the authentication range from a concatenated N-dimensional feature vector to a single-dimensional (gradient) vector space by representing the physical layer authentication as a linear system, leading to reduced authentication cost. By recasting the physical layer authentication learning (training) goal as a convex issue, an adaptive algorithm based on kernel least-mean-square is proposed as an intelligent procedure for learning and tracking numerous attribute modifications, thereby improving the authentication efficiency.

Another different technique based on the authentication interval log has been proposed in [143,144,145]. The data collection (Access logs) goes through feature selection and the ML mechanism (KNN, RF, MLP, and Gradient Boosting) to identify the authentication policy. A small amount of information was reported in [144], while [143] showed the authentication accuracy, and [145] showed how to use protocol access history to create user authentication models. However, these proposed methods are valid if the WSN nodes move regularly all the time.

**Table 7:** Summary of reviewed ML algorithms in the authentication.

Refs.	ML Technique	Processing Cost	Advantage	Accuracy	Limitations
[141]	LTSM	Moderate	Improved performance accuracy for long-term fault signals	99.5%	Centralization of authentication Not suitable for massive WAN nodes
[21]	Gradient algorithm + DNN	Low	Improved authentication rate through reducing training time	91%	Centralization of authentication
[22]	Channel information + ML	Low	Improved authentication rate by using ε-greedy strategy	99.8%	Not effective for large networks
[142]	kernel least-mean-square	High	Improved authentication rate by using reducing N-dimensional vector to a single-dimensional vector space	97.5%	Do not take into account the parameters of channel weakness
[143]	Various ML algorithms	Moderate	Improved performance accuracy through tracing WSN node behavior	96%	It consumes more memory Increase searching time
[145]	Various ML algorithms	Moderate	Improved performance accuracy through WSN node history	97.5%	It consumes more memory Increase searching time

### 5.3. ML-Based WSN Diversified Security

In this section, we discuss the role of ML algorithms in wireless sensor network security in different areas other than those discussed in the previous subsection, including the man in the middle, espionage, and selective forward.

In [146], the authors used the neural network method, which consists of three neurons (devices, sensing, and delay); five hidden neurons at three levels were also used. Through those inputs to the packets on the network, the proposed algorithm monitors the health of each node. If the values deviate from the expected value, this indicates false information or the presence of a man in the middle of the attack. In another technique that uses a ML technique to identify WSN devices, the authors in [147] proposed a new model to classify newly assigned devices in the home or office as trustworthy, stringent, or limited. The gateway is responsible for supervising the traffic generated by newly assigned devices and generating device fingerprints that are sent to an IoT security service provider, which then identifies the device based on its type and traffic using a ML classification model. Furthermore, the authors in [148] used a ML method to distinguish between WSN and non-WSN devices based on data traffic. For categorization, sessions and features extracted from each device are being used.

In addition, the approach described in [26] used a ML technique to determine whether a benevolent node had turned malevolent. Bio-inspiration can also be used as an immune system to counteract the effects of malevolent nodes. To begin, the k-means algorithm divides the data into two sets: normal and defective. After that, SVM is used to generate a decision block with three regions: normal, defect, and critical at the borders. The mean and standard deviation of the WSN node supplied by the SVM dataset are determined using an anomaly detection technique. The immune system is then engaged after an anomaly is discovered. Virtual antibodies are created, and malevolent nodes are eventually killed, similar to biological processes.

Regarding WSN privacy and safety, the authors in [149] evaluated ML algorithms for interference detection that focus entirely on the analysis of samples received in-phase (I) and Quadratic phase (Q). Mitigation measures can be used once an intrusion has been identified, highlighting the need for interference detection. The Random Forest classifier was chosen because it comprises a huge number of individual decision trees that work together as an ensemble. Next, the same authors in [74] investigated the performance of Random Forest and SVM classifiers on WSN channel identification. The authors extracted data features from the samples received in I and Q and then collected other data from wired devices (without interfering). In the next step, the authors train the data collected from I and Q on ML algorithms and then evaluate them for signal-free, valid signal, and jamming signal situations. Finally, the outputs of these classifiers were compared to the data collected from wired (no interference).

Table 8 provides an analysis of the reviewed studies in ML-based WSN diversified security.

## 6. Discussion and Open Issues

Each of the suggestions in the previous section has the characteristics to improve one or more parts of the wireless security process for WSNs. The reviewed studies provided good information about the differences between the types of ML algorithms in terms of outputs and implementation. Moreover, the ML algorithms in some domains of the security requirements, such as availability, provide amazing outputs by monitoring errors, congestion, and identifying malicious packets. In addition, in the process of providing authentication using the physical layer (the first layer) and analyzing the signal channel, ML algorithms also provided good outputs. However, the gap remains, where the ML algorithms are unable to provide all the security requirements in WSN. Therefore, the ML algorithms used, and their methods must comply with the security requirements set by public security agencies (e.g., IEEE, IETF).

**Figure 12:**
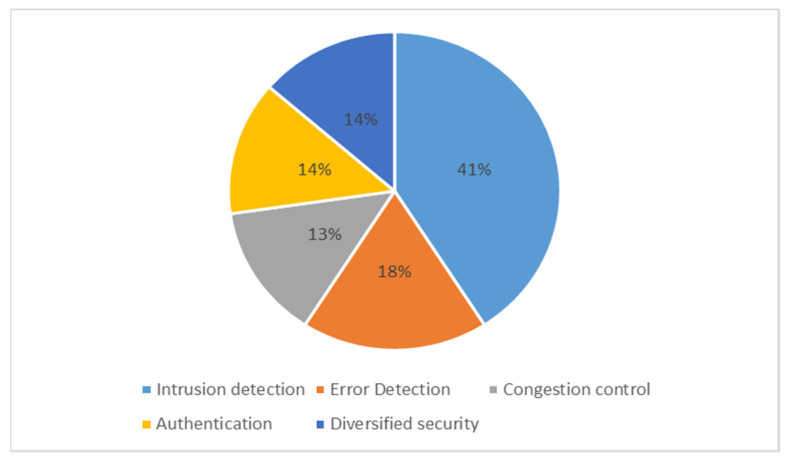
Statistical analysis for the reviewed ML algorithms in WSN security.

Figure 12 shows the results of the statistical analysis on WSN security implementations that were performed on the reviewed ML algorithms. The analysis shows the type of security implemented in WSNs and the percentage of ML algorithms used in each type.

As shown in Figure 12, 41% of the reviewed studies used ML algorithms for intrusion detection. It is closely followed by 18% for error detection. The remaining 14% converge for follow-up studies. This is due to the cost of ML algorithms on hardware (devices), as well as their need for training processes. Moreover, most of it was applied to the part of maintaining network availability and the difficulty of applying it to both confidentiality and integrity. In the next points, we discuss the preceding studies and some open issues that need in-depth research regarding the use of ML algorithms in the security of WSNs of three types (confidentiality, integrity, and availability) and suggest some solutions to these challenges.

### 6.1. Location of the ML Training Process

One of the most important issues for this type of network is where to implement ML in the training process, as it is scattered rather than centralized, and all its embedded devices are equal in CPU and energy. Therefore, in most studies that have used ML techniques, we obtain ambiguity about how these algorithms were performed in the WSN environments. Most existing works have improved the accuracy of identifying the attack or malicious node [77,78,120], but it is not clear where to train those algorithms. Furthermore, we do not realize how much power these embedded devices lose in executing training operations or detecting packets in DoS detection.

**Figure 13:**
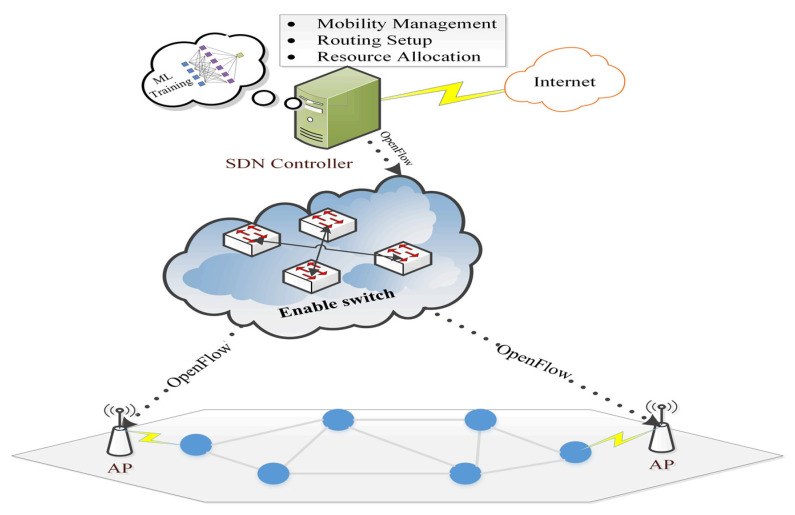
SDN architecture in implementation of ML algorithms on WSNs.

Other authors have taken advantage of SDN technology [150] by performing the training operations in the controller and then sending their outputs (training process) to the WSN nodes via the SDN-Switches [124,126]. It is considered a successful and good idea, but it also needs to develop special protocols to deal with these particles. Technologies, such as SDN, Multi-access Edge Computing (MEC) [151], and Network Function Virtualization (NFV) [152] make networks programmable, allowing for faster service management and flexibility in data updating. This technology divides large networks into a group of small networks that are independent in management and linked to neighboring networks with the same technology (SDN) [153]. Small networks are also centralized in management and are managed by a device called an SD-Controller, which is responsible for managing both the network equipment and all users belonging to that network. Therefore, if we assume that all network sensors belong to the same SDN, as shown in Figure 13. The possibility of transferring the data training processes of ML algorithms from the WSN nodes to the SDN-Controller becomes possible. In the process, we will keep the WSN’s security up-to-date at no cost. For example, [127] has taken this step; however, there are a set of updates that SDN-Switch needs to avoid packet congestion in inspections.

From Figure 13, we can see that the SDN consists of a central device called the SD-Controller, and a network device called the SD-Switches. SD-Switches Connect to wireless devices (WSN nodes) through the AP. Data are transferred between these devices using a protocol called OpenFlow [154]. It is responsible for passing data between the network equipment and the SD-Controller, as well as receiving updates from the SD-Controller. Therefore, this proposal represents an ideal solution to the problem of where to train machine learning algorithms.

Furthermore, other open issues can be taken advantage of, such as all WSN nodes in this type of network being equal in terms of tasks and nature of work. Therefore, another solution is to develop a clustering principle between nearby WSN nodes to share training processes for ML algorithms. This is done by developing special protocols for dealing with ML algorithms, and sending and receiving data as well as output.

### 6.2. Lightweight ML Algorithms

In addition, we have also realized that a group of authors has implemented complex ML algorithms to improve the efficiency of accuracy, without paying attention to the hardware requirements it needs. Moreover, as mentioned earlier, the ML algorithms are divided into several categories, they are also accompanied by algorithms that help them to reduce training time and raise the level of accuracy. Therefore, it is possible to create a hybrid type (ML approach) that is lightweight and suitable for working on such types of embedded devices. On the other hand, it is possible to develop the ability of WSN nodes to become able to distinguish between types of ML algorithms and automatically select the appropriate one based on several factors, including data type, data volume, and remaining power. This is also one of the open issues in the future that researchers should try to improve.

In addition, none of the studies reviewed in the areas of application of ML to support the security of WSNs addressed the use of reinforcement learning [155] or transfer learning [156]. The future of machine learning tends to use these technologies. Reinforcement learning is experience-based learning rather than training on the data, and transferal learning is based on pre-trained models. So, in open issues, it is possible to try both methods along with SDN.

### 6.3. Privacy Concerns

As mentioned earlier, all WSN nodes are equal in terms of CPU, energy, and tasks. Furthermore, the environments in which these embedded devices operate are also mobile, thus the interrelationship between a node’s communication with its peers is constantly changing. The node’s current authentication mechanism is useless with all this change. Furthermore, what the authors have done by exploiting the first layer (physical layer) in the authentication process and using ML algorithms to help with the authentication process is also a good option that reduces synchronization and acknowledgment as well as saves nodes energy. However, one of the points that are considered an open issue is the privacy condition. It is an open field to research ways to keep the privacy of sensors from being hacked by peers, whether intentionally or unintentionally.

### 6.4. Trust Domain

One of the issues that could enhance the security of wireless sensor networks is the development of trust between the WSN nodes [157]. This is also done by observing the behavior of a WSN node, then based on ML algorithms, assigning the trusted moniker “Reliable” to nodes with a good reputation among their neighbors. This is accomplished by improving the management of WSNs so that each node’s behavior may be analyzed and the results shared with other nodes. ML algorithms will also play an important role in analyzing the behavior of adjacent nodes in collaboration with the SD-Controller.

## 7. Conclusions

The wireless sensor network is intended to carry out routine tasks, such as data collection and monitoring in a range of environments. The Internet of Things has led to a growing dependence on this sort of network due to its simplicity, ability to fulfill specialized activities, and low cost. The rising proliferation has been followed by several issues, one of which is the security issue. In comparison to traditional networks, fundamental security needs in wireless sensor networks are difficult to achieve due to limited CPU and power. As a result, novel approaches to this problem are required. In an attempt to overcome this challenge, various investigations have used ML methodologies. Adapting ML algorithms to WSNs is also fraught with difficulties. In this paper, we presented an illustrative study of the wireless sensor network infrastructure, its environment, applications, operating process, and security challenges associated with it. Then we reviewed the ML algorithms that were used in them, and we conducted an analytical study of the recent studies that worked to improve security in WSNs using ML algorithms. We also showed the pros and cons of each study. Subsequently, this paper worked on showing future solutions that could help exploit the algorithms of machine learning in the field of security for WSNs due to its promising future in this field.

Through the statistical analysis of the use of ML algorithms in the security of WSNs, intrusion detection and error detection are the most common uses. One of the most essential and optimal choices for expanding the usage of ML algorithms in additional security domains is to employ SDN technology. It may be used to improve WSN node efficiency while also lowering the cost of usage.

## Figures and Tables

**Table 1:** Attacks in security policies.

Security Infrastructure	Attacks
Confidentiality	Hole, Sybil, Spoofing, Session hijacking, Repudiation, Selective forwarding, Spoofing
Integrity	Eavesdropping, traffic analysis, Selective forwarding, Spoofing
Availability	DoS, Exhaustion, Jamming, Collision, Unfairness

**Table 2:** WSNs protection techniques.

Security Infrastructure	Attacks
Confidentiality	Encryption
Integrity	Digital signature, MAC
Availability	Traffic control, redundancy, Rerouting
Non-repudiation	Digital certificate

**Table 3:** ML challenges in WSN security.

No.	Challenges
1.	Accurate real-time predictions
2.	The use of ML does not cover all the security requirements of WSNs
3.	Outputs are approx.

**Table 5:** Summary of reviewed ML algorithms in error detection.

Refs.	ML Technique	Processing Cost	Error Detected	Accuracy	Limitations
[23]	SVM, KNN, and RNN	Relative	Offset, gain, stuck-at, and out of bounds	97%	Calculating the reliability of the decision is complex
[128]	hidden Markov model + Neural networks (NNs)	high	Random, drift, and spike	96%	Training speed is slow
[129]	SVM	Low	Negative alerts	99%	Does not consider the load management between nodes
[130]	SVM	High	Fault WSN nodes	98%	Not suitable for large networks
[131]	SVM + principal component analysis	High	Fault WSN nodes	99%	complexity is high
[24]	Bayesian	High	Fault WSN nodes	70%	Bayesian increases the complexity of the WSN devices
[25]	Bayesian	High	Fault WSN nodes	100%	It takes more time to detect due to the use of two different detection systems
[132]	KNN	Moderate	Fault WSN nodes	99%	Not cover continuous change in WSN topology

**Table 6:** Summary of reviewed ML algorithms in congestion control.

Refs.	ML Technique	Processing Cost	Control Policy	Detection Criteria
[17]	Fuzzy logic	Low	Queue management	Buffer occupancy
[133]	Fuzzy logic	moderate	Queue management	buffer occupancy
[18]	Fuzzy logic	High	Traffic control	Buffer occupancy
[19]	Heuristic and Fuzzy logic	High	Traffic control	Channel load
[134]	K-mean, Firefly, and ant colony	High	Traffic control	Packet service time
[135]	Fuzzy logic	Low	Traffic control	Buffer occupancy

**Table 8:** Summary of reviewed ML algorithms in WSN diversified security.

Refs.	ML Technique	Processing Cost	Attack	Accuracy	Limitations
[146]	ANN	High	Man in the Middle	99%	It needs huge data sets
[147]	Random Forest	Low	Traffic monitoring (identification)	96%	Not expandable
[148]	Binary classifier	Low	Traffic monitoring (identification)	95%	Centralization of classification
[26]	k-mean + SVM	Moderate	Malicious node	NA	Centralization of classification
[149]	Random forest	Low	Privacy	NA	Require large memory for storage
[74]	Random Forest + SVM	Moderate	Channel identification	NA	Not effective for large networks

## Data Availability

Not applicable.
